# Isolated greater omentum solid mass, a rare manifestation of hydatid disease; A Case Report and Review of Literature

**DOI:** 10.22088/cjim.14.2.386

**Published:** 2023

**Authors:** Ali Jangjoo, Sina Norouzi Asl, Yeganeh Azadmanesh, Tooraj Zandbaf

**Affiliations:** 1 Surgical Oncology Research Center, Mashhad University of Medical Sciences, Mashhad, Iran; 2 Department of Emergency Medicine, Razavi Hospital, Mashhad, Iran; 3 Department of General Surgery, Faculty of Medicine, Mashhad Medical Sciences, Islamic Azad University, Mashhad, Iran

**Keywords:** Hydatid disease, Hydatid Cyst, Echinococcus Granulosus, Greater Omentum, Echinococcosis, Case Report.

## Abstract

**Background:**

Echinococcus granulosus causes hydatid disease, which is found in various countries of the world, including Iran. The liver and lungs are prevalent involved structures in hydatid disease. One of the least common sites in hydatid disease seems to be the omentum. Seven cases of mesenteric, diaphragmatic, omental, pelvic, and retroperitoneal hydatid cysts have been reported in Iran within last 20 years. The appearance of hydatid disease as a primary mass in the greater omentum without hepatic involvement is very rare and no similar case was introduced in Iran in our searches.

**Case Presentation:**

Our patient was a 33-year-old woman who underwent a diagnostic laparoscopy due to abdominal pain and an abdominal mass. During laparoscopy, there was a solid mass with a size of about 10 × 5 cm in the greater omentum that was resected. The histopathological examination of the mass showed the hydatid disease.

**Conclusion:**

The hydatid cyst can appear anywhere on the body, and no part of the body is guarded. Since these uncommon locations often cause nonspecific symptoms, the hydatid cyst should be included in the differential diagnosing of omental cysts, particularly in endemic countries like Iran.

Hydatid disease is an infectious disease caused mainly by Echinococcus granulosus' larval stage and is still a major public health issue around the world (1). It is found in most Mediterranean sheep-raising countries, including Iran. Hydatid disease mostly affects the liver and lungs, but it may also affect other parts of the body. Peritoneal hydatid cysts, whether primary or secondary, are a rare but important occurrence of the disease (2). Peritoneal hydatid disease is a rare condition that occurs in roughly 13% of cases and most of these cases are linked to earlier operations for liver hydatid disease. It could be linked to a mechanical burst of hepatic or splenic hydatid infection in some situations. Primitive peritoneal disease without the occupancy of any other structure is extremely uncommon (3). These rare positions include the peritoneum, omentum, and mesentery, which can make diagnosis difficult and cause treatment delays. Surgical excision, with or without adjuvant therapy, albendazole, and scolicidal agents, has proven to be the most effective treatment option (4). We report a 33-year-old woman who underwent diagnostic laparoscopy due to an abdominal mass. 

In our searches, this patient seems to be the first case of hydatid disease to be reported as a greater omentum solid mass without hepatic involvement in Iran. For this reason, we would like to present our clinical experience with this rare patient. This study was approved by the Research Ethics Committees of Islamic Azad University- Mashhad Branch (IR.IAU.MSHD.REC.1400.115). Our case has been reported by the SCARE guidelines.

## Case Presentation

**Figure 1:**
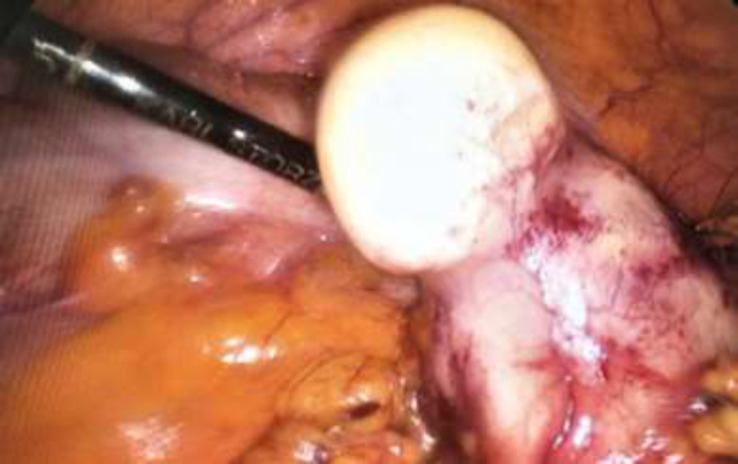
Laparoscopic View of Greater Omentum Solid Mass

**Figure 2:**
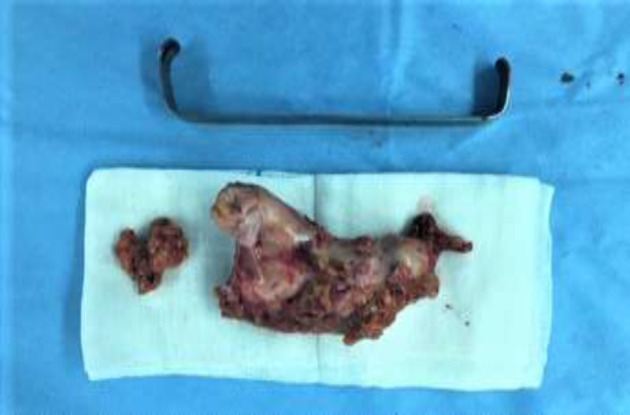
Macroscopic View of Greater Omentum Solid Mass

A 33-year-old woman with no history of the previous disease was referred to a general surgery department with abdominal pain and an abdominal mass. She has been suffering from abdominal pain for about 4 months and has been feeling an abdominal mass since a month ago. During the physical examination, a mass was felt along the umbilicus and the mid-clavicular line on the right side of the abdomen, 

which was slightly tender. Routine lab tests and colonoscopy were normal. An abdominal CT scan showed an increased thickness in the terminal ileum while other abdominal organs reported normal. The patient was nominated for diagnostic laparoscopy. After general anesthesia, a 10 mm port was inserted through the umbilicus by the open manner. Next, the pneumoperitoneum was established, a 30-degree laparoscope was inserted into the abdomen, and the whole abdomen was explored. A solid mass with a size of about 10 × 5 cm was seen in a greater omentum (figure 1). Two 5 mm ports were put in the subxiphoid area and the RLQ. There was no other pathology in abdominal exploration. Finally, the mass was completely resected from the omentum and removed from the umbilical incision with an endo bag (figure 2). On post-operation day two, the patient was discharged home without any troubles. Histopathological examination of the mass showed a Hydatid disease. 

## Discussion

In endemic areas, disease incidence rates can exceed 50 per 100,000 individual-years, with an outbreak of 5% to 10%. Iran is one of the locations designated by the World Health Organization as a hyper-endemic area due to a high proportion of the population's intimate association with animals, traditional beast farming, and subsequent communication with infection origins. In different locations of Iran, the prevalence of hydatid disease has been documented from 1.1% to 13.7%. In Khorasan Razavi Province, the prevalence of hydatidosis was 1.44 per 100,000 people. The age category of 21-40 years old had the largest proportion of hydatidosis (39.2%). The majority of clinical symptoms in hydatidosis were abdominal pain (42.3%), followed by hepatomegaly (27.4%), chest pain (21.6%), and cough (8.7%) (5). Similarly, our patient's age was consistent with the results of this study. In addition, our patient complained of abdominal pain but did not have hepatomegaly and other symptoms mentioned in this study. Cystic echinococcosis is a condition that affects young adults, with the age of 30–40 years old. The parasite load, location, and the size of the cysts all influence the severity of symptoms. Echinococcus has a complicated taxonomy. Four main species of Echinococcus have been recognized. Cystic echinococcosis, alveolar echinococcosis, and polycystic echinococcosis are created by Echinococcus granulosis, Echinococcus multilocularis, and either Echinococcus vogeli or Echinococcus oligarthus respectively. Echinococcus mulitilocularis is the most infectious of all, while Echinococcus vegeli and Echinococcus oligarthus are not common (5,6).

Peritoneal hydatidosis is a common side effect of surgical treatment for a hepatic or splenic hydatid cyst. Primitive peritoneal hydatidosis is unusual, with almost about 2% of all instances of abdominal hydatid diseases. It's unclear what causes primary peritoneal involvement. Dissemination into the peritoneal cavity can occur by lymphatic or systemic circulation. The third option is that a superficial hepatic hydatid cyst ruptured spontaneously, causing considerable peritoneal involvement. The peritoneal hydatid cyst produces no symptoms in most cases, and the diagnosis was created when the patient gets an ultrasound for an irrelevant reason (3,7). In many situations, disease symptoms are missing, and infection is discovered only by chance during imaging investigations. Hydatid infection treated with albendazole and surgical operation, or albendazole with PAIR (Puncture, Aspiration, Injection of scolicidal agent, and re-aspiration) to remove the cyst safely. Because of the high rate of recurrence of hydatid disease after therapy with surgical operation or albendazole, a multidisciplinary strategy was suggested, as is an ongoing pharmaceutical treatment for minimum three months after surgery. Albendazole, an oral benzimidazole antihelmintic, is the medication of choice for echinococcal disease treatment and should be employed in patients with inoperable primitive and peritoneal hydatid cysts. It can control a recurrence of echinococcal infection caused by cyst contents spilling out after surgery or cyst draining. The medication is offered in two divided doses of 10 to 15 mg/kg/d in four-week cycles, with no pharmacological therapy for two weeks. Relying on the intensity of the illness or the recovery of the patients, this regimen is consecutive for numerous periods (8). Due to the lack of diagnosis before surgery, it was not possible to use PAIR as well as albendazole. However, after surgery and based on pathological findings, the patient was treated with albendazole.

Echinococcosis can potentially affect any organs in the body. The liver, followed by the lungs are organs that can be affected. 90% of occurrences of echinococcosis are found in these two organs (6,9). Given the observed variations in hydatid cyst sequences, it appears that hydatid cyst replacement in organs other than the liver and lungs is highly associated with genotypes and intra-genotypic characteristics. It is assumed that less prevalent genotypes in every unique geographic region, might be more correlated with the host’s uncommon organs' placement. G2 and G3 genotypes are more frequent in rare organs (10). Utpal presented a 56-year-old woman who had been suffering from acute right lower abdomen discomfort, nausea, vomiting, and a fever for the past five days.

 An abdominal examination revealed a painful solid mass measuring 5 cm x 3 cm in the right lower quadrant with restricted mobility. Further studies were carried out to rule out the possibility of an appendicular mass. Abdominal ultrasonography revealed a multiseptated cyst with a honeycomb appearance, which was suggestive of Hydatid disease. The cyst was discovered in the appendicular mesentery during laparotomy. The procedure included an excision and an appendectomy (7). Although the onset of the disease as a mass is like ours, the course of the disease in our patient was not acute and with abdominal symptoms. In addition, the location of the mass in our patient was in the greater omentum, while in the above patient, the cyst was in the appendicular mesentery.

Mejri et al. presented a 52-year-old female patient with steadily growing abdominal distension over 10 months and dull abdominal pain. Multiple enormous cysts were found throughout the peritoneal cavity, liver, and mesentery on a CT scan. Serology for hydatids was extremely positive. A two-step surgery was completed, allowing for the removal of the peritoneal cysts as well as a partial cystectomy for the hepatic cyst (11). In our patient, contrary to the reported case, there was only a single mass in the greater omentum and the liver was free of disease. In addition, the surgery was performed in one step. Seven cases of peritoneal hydatidosis were described by Acharya et al. One of them had diffused primary peritoneal echinococcosis, which is a rare occurrence, while the others were caused by hepatic or splenic diseases. They were given an antihelminthic treatment before surgery, and then removal of the peritoneal cysts, as well as de-roofing and omentoplasty for the hepatic lesions, and splenectomy for the splenic hydatid was done. All patients were offered three months of albendazole during follow-up which is accomplishing healthful (12).

Seven cases of mesenteric, diaphragmatic, omental, pelvic, and retroperitoneal hydatid cysts have been identified in Iran within last 20 years (13). Assessment of the disease's epidemiological and clinical characteristics can assist health policymakers in focusing on the community's most important public health issues and evaluating the efficacy of control and prevention measures aimed at limiting the parasite's spread and transmission in people. The spread of hydatidosis, as a major ignored disease, should be thought-out by public health policymakers. Furthermore, in high-risk populations, educational programs to improve disease symptom awareness and infection source identification are required (5). The appearance of hydatid disease as a mass in the greater omentum without hepatic involvement is very rare and no similar case was introduced in Iran in our searches. The hydatid cyst can appear anywhere on the body, and no part of the body is guarded. Since these uncommon locations often cause nonspecific symptoms, the hydatid cyst should be included in the differential diagnosing of omental mass, particularly in endemic countries like Iran.

## References

[1] Sekmenli T, Koplay M, Sezgin A (2009). Isolated omental hydatid cyst: clinical, radiologic, and pathologic findings. J Pediatr Surg.

[2] Ghafouri A, Nasiri S, Far AS (2010). Isolated primary hydatid disease of omentum; report of a case and review of the literature. Iran J Med Sci.

[3] Kumar KLS (2009). A case of primary peritoneal hydatidosis. Med J Armed Forces India.

[4] Geramizadeh B (2017). Isolated peritoneal, mesenteric, and omental hydatid cyst: A clinicopathologic narrative review. Iran J Med Sci.

[5] Khazaei S, Rezaeian S, Khazaei Z (2016). Epidemiological and clinical characteristics of patients with hydatid cysts in Khorasan Razavi Province, from 2011 to 2014. Iran J Parasitol.

[6] Gessese AT (2020). Review on epidemiology and public health significance of hydatidosis. Vet Med Int.

[7] De U (2009). Primary abdominal hydatid cyst presenting in emergency as appendicular mass: a case report. World J Emerg Surg.

[8] Sabouni F, Ferdosian F, Mamishi S (2010). Multiple Organ Involvement with Hydatid Cysts. Iran J Parasitol.

[9] Shahriarirad R, Erfani A, Eskandarisani M (2020). Human cystic echinococcosis in southwest Iran a 15-year retrospective epidemiological study of hospitalized cases. Trop Med Health.

[10] Shafiei R, Ghatee MA, Jafarzadeh F, Javanshir Z, Karamian M (2019). Genotyping and phylogenetic analysis of unusually located hydatid cysts isolated from humans in north-east Iran. J Helminthol.

[11] Mejri A, Arfaoui K, Ayadi MF, Yaakoubi J, Aloui B (2020). This is what we call peritoneal hydatidosis. Int J Infect Dis.

[12] Acharya AN, Gupta S (2009). Peritoneal hydatidosis: a review of seven cases. Trop Gastroenterol.

[13] Geramizadeh B (2013). Unusual locations of the hydatid cyst: A review from Iran. Iran J Med Sci.

